# Autoantibodies against the chromosomal passenger protein INCENP found in a patient with Graham Little-Piccardi-Lassueur syndrome

**DOI:** 10.1186/1740-2557-4-1

**Published:** 2007-01-12

**Authors:** Beatriz Rodríguez-Bayona, Sandrine Ruchaud, Carmen Rodríguez, Mario Linares, Antonio Astola, Manuela Ortiz, William C Earnshaw, Manuel M Valdivia

**Affiliations:** 1 Servicio de Inmunología, Hospital Puerta del Mar, 11109 Cádiz, Spain; 2 Dermatología, Instituto Social de la Marina, Delegación Provincial de Cádiz, Cádiz, Spain; 3 Wellcome Trust Centre for Cell Biology, School of Biological Sciences, University of Edinburgh, Edinburgh EH9 3JR, UK; 4 Departamento de Bioquímica y Biología Molecular, Facultad de Ciencias, 11510 Puerto Real, Cádiz, Spain

## Abstract

**Background:**

Graham Little – Piccardi – Lassueur (GLPL) syndrome is a rare dermatosis characterized by scarring alopecia, loss of pubic and axillary hair, and progressive development of variously located follicular papules. We report a first case ever of an autoimmune response in a patient suffering from GLPL syndrome.

**Methods:**

Immunofluorescence and immunoblot analysis were used in a variety of cell cultures including human, monkey, hamster, mouse and bovine cells to analyze the presence of autoantibodies in a GLPL patient.

**Results:**

The autoimmune serum showed a pattern of centromere and spindle microtubule staining resembling that of the chromosomal passenger protein complex. By using a complex of proteins expressed in baculovirus, immunoblot analysis demonstrated that the INCENP protein is a major autoantigen in this patient with GLPL syndrome.

**Conclusion:**

An autoimmune response in GLPL syndrome is reported against the INCENP centromere protein. The occasional development of autoimmunity in GLPL patients could serve as a test in continuing efforts to detect this disease and for a more directed therapy based on the autoantigen response.

## Background

The presence of cicatricial alopecia on the scalp, keratosis pilaris on the trunk and extremities, and non-cicatricial hair loss in the pubis and axillae was first described by Piccardi in 1914 and a year later by Graham Little in a patient sent by Lassueur. The GLPL syndrome is considered a form of follicular lichen planus (LP), characterized by lichenoid dermatosis, keratosis pilaris and progressive cicatricial alopecia of the scalp [1]. All the clinical manifestations need not to be present simultaneously. Association with androgen insensitivity syndrome was also recently described for GLPL Syndrome.

A familial case of occurrence in a mother and her daughter was recently described with a HLA-DR1 in both patients [2]. Topical or systemic corticosteroids, retinoids or PUVA therapy are the treatments usually proposed and these offer partial and temporary benefits [3]. More recently the effectiveness of cyclosporin A was described for the first time [4].

Immunofluorescence (IF) analysis is a common clinical procedure used in studying the development and behavior of several chronic diseases. Based on clinical features and histological findings, we decided to perform an initial IF test in a woman patient suffering GLPL syndrome. Autoantibodies were found to recognize a mitotic-associated antigen which behaves like the previously-described chromosomal passenger complex protein [5]. Our findings represent the first report to date of an autoimmune response in GLPL syndrome and this work, also allow us to describe the first autoantibody directed to INCENP protein in humans. Further studies of the significance of autoimmunity in GLPL patients will follow in the future.

## Materials and methods

### Human autoimmune serum

A 45 year old woman with alopecia in frontal area with follicular hyperkeratosis and other clinical features was diagnosed as suffering GLPL syndrome. Initial clinical test by IF on standard human HEP2-fixed cells indicated positive antinuclear autoantibodies (ANAs) and specific midbody localization (data not shown). Anti SM, SSB, SSA, CENP-B, Jo-1, ribosomal P, and histones were also initially determined negative by immunoblots.

### Immunofluorescence analysis

Culture cells from hamster (CHO), monkey (CV1), mouse (3T3) and bovine (MDBK) were grown on coverslips at 37°C in Dulbecco's modified Eagle's medium (Gibco, Life Technologies Inc.) containing 7% fetal calf serum. Cells were fixed in methanol for 10 min. at – 20°C for autoantibody staining. The human GLPL serum was used at 1:400 dilution in PBS for 45 min at 37°C followed by 4 × 5 min time washes in PBS. FITC-conjugated goat antihuman-Ig (Santa Cruz Lab. CA, USA) was used as a second antibody at 1:100 dilutions in PBS for 45 min at 37°C. This was followed by 3 × 10 min washes in PBS. Cells were counterstained for Hoechst and mounted in PBS:glycerol 9:1. Staining was observed in a Zeiss epifluorescent microscope and immunofluorescence images were recorded by a CCD Spot Camera (Diagnostic Instruments Inc. USA). For double indirect immunofluorescence microscopy, antibodies against Aurora B (Abcam), INCENP (rabbit 1186), alfa-tubulin (Aldrich) and secondary antibodies coupled to FITC, Texas red or Cy5 (Jackson ImmunoResearch Laboratories) were used in human U2OS cells following methods previously described [6]. DNA was visualized with 0.5 ug/ml DAPI and image stacks were captured using an Olympus microscope controlled by DeltaVision SoftWorx (Applied Precision). Images were deconvolved and image stacks quick-projected.

### Cell extract, recombinant antigen preparation and immunoblot analysis

Whole cell extracts were prepared from HeLa culture cells blocked with colcemid by lysis in SDS Laemmli buffer. Three samples of 10/20/40 micrograms of protein were loading on a gel. The cDNAs and baculovirus constructs for Aurora B, Surviving, and INCENP have been previously described [7]. Insect Sf-9 cells were infected with appropriate combinations of the constructs and complexes containing GST-Aurora B, GST-INCENP, and His-Survivin were purified on glutathione sepharose beads (Amersham Biosciences) [8]. Cell extract and expressed proteins were loaded in a 10% SDS-acrylamide gel and transferred to nitrocellulose membranes (Millipore) in the presence of Tris-HCl-glycine-SDS buffer [9]. The membrane was blocked in 5% dried milk powder in PBS-0.1% Tween for 2 hours at room temperature followed by incubation with the human autoimmune GLPL serum at 1:200 dilution in PBS at room temperature for 12 hours. Several washes in PBS-Tween were followed by reactivity with a peroxidase-conjugated goat-anti human Ig (Sigma Chemical Co, USA) diluted 1:3000 in PBS for 2 hours at room temperature. The membrane was washed again for 3 × 10 min period in PBS-Tween and developed with 4-chloro-1-naphthol (Sigma Chemical Co, USA) in PBS-methanol-H_2_O_2 _until the color bands appeared. Rabbit specific serum to INCENP protein [6] was assayed as a control on blots with recombinant passenger protein complex.

## Results and Discussion

### Cellular distribution of the GLPL syndrome autoantigen

**Figure 1:**
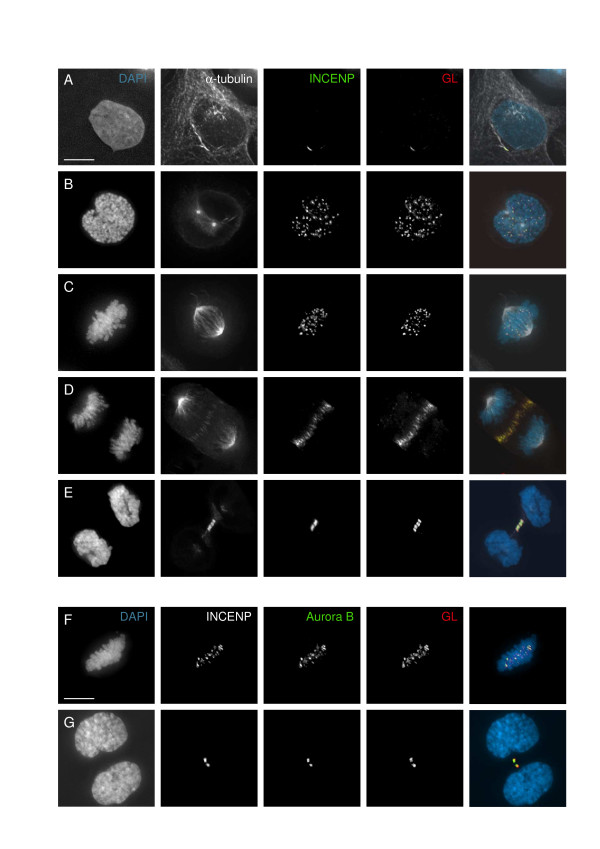
*Immunofluorescence of human cells with Graham Little autoimmune serum*. (A-E) localization of GL autoantigen in red and INCENP in green during interphase (A), prophase (B), metaphase (C), anaphase (D) and telophase (E). Anti-tubulin staining is shown in white and merge images with DNA in blue. GL autoantigen co-localizes with passenger protein INCENP in human U2OS cells. (F-G) Co-localization of GL autoantigen in red with INCENP in white and Aurora B in green at metaphase (F) and telophase (G). The merge images represent GL, Aurora B and DAPI. Scale bars 10 micro m.

**Figure 2:**
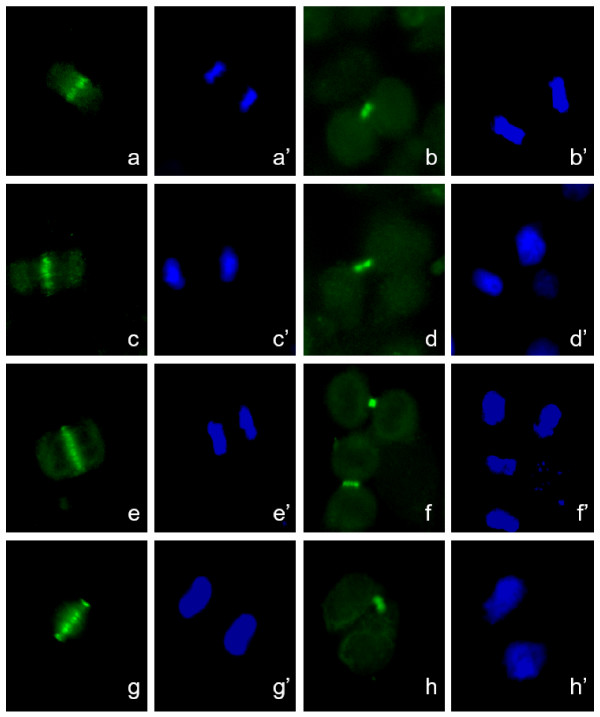
*Graham Little human autoantibody recognizes chromosomal passenger proteins in different mammalian cell lines*. Staining of cells in anaphase (a, c, e, g) and midbody in cytokinesis (b, d, f, h) is shown for hamster (CHO in a-b), mouse (3T3 in c-d), bovine (MDBK in e-f) and monkey cells (CV-1 in g-h). The autoimmune serum reacts with conserved epitopes of passenger proteins in diverse species. Nuclei staining by Hoechst is shown in a'-h'.

In a routine clinical test by IF on standard HEP2 cells, the Graham Little-Piccardi-Lassueur serum showed a midbody staining reminiscent of chromosome passenger proteins (data not shown). To characterize in detail the human autoimmune response, U2OS culture cells were assayed by IF with GLPL serum showing a typical pattern of staining in mitosis and interphase cells. During prophase the autoantigen was found on the condensing chromosomes, gradually becoming concentrated at the centromeres [Figure 1B, GL]. This was evident in metaphase, where the autoantigen was localized at the centromeres apparently in all chromosomes [Figure 1C, GL]. During anaphase and telophase the staining was associated with microtubule bundles in the central spindle and in the cleavage furrow as indicated by double staining with anti-tubulin antibody [see details in white and red in Figure 1D,E]. These IF patterns in human cells unequivocally suggested to us that the GLPL serum presented reactivity against a chromosomal passenger component and this represented an autoimmune response in the patient. This was corroborated by double IF staining with specific anti-INCENP serum (green in Figure 1A–E). Further, to clearly show that the GLPL autoantigen localizes like a Chromosomal Passenger in human U2OS cells, four color staining was performed with anti-Aurora B serum (green in Figure 1E–F). Using this technology, unequivocally we can clearly show in Figure 1, that the GLPL autoantigen (red) co-localize with INCENP (white) and Aurora B (green) proteins. The IF pattern observed was not restricted to human cells, as shown in Figure 2. Culture cells of different species showed a similar if not identical IF staining pattern to those of human cells during all the cell cycle stages. Some examples of IF localization observed in hamster [Fig. 2a,b], mouse [Fig. 2c,d], bovine [Fig. 2e,f] and monkey cells [Fig. 2g,h] are presented. Identical IF patterns were found for the GLPL autoantigen association to microtubule inter-chromosomal bundles and midbody during cytokinesis in all the cell lines assayed. This corroborated our initial hypothesis that the autoimmune response in GLPL patient targeted several components of the highly-conserved Chromosomal Passenger complex already described from yeast to humans [10].

### INCENP protein is a major autoantigen in a GLPL syndrome patient

**Figure 3:**
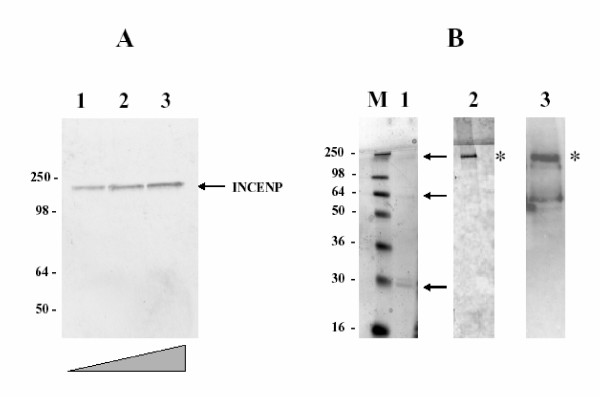
*Identification of INCENP as the Graham Little human autoantigen by western blot analysis*. In **A: **A HeLa cell extract was used by immunoblot against the autoimmune GL serum to show reactivity with a 135 kD polypeptide. Increased amounts of cell extracts are shown in lanes 1–3. In **B: **a recombinant complex of GST-INCENP (upper arrow), GST-Aurora B (middle arrow) and His-Survivin (lower arrow) shown in lane 1, are blotted against the GL serum (lane 2). Only the INCENP component reacts by blot with the human autoantibody (asterisk in lane 2). The recombinant proteins in lane1, were assayed in a blot control against a specific anti-INCENP serum as shown in lane 3. As marked by asterisk in both lanes 2 and 3, the INCENP component is a major autoantigen recognized by GL human autoimmune serum.

The next step in our study was to identify the GLPL autoantigen by performing immunoblot analysis. First, we used human whole cell extracts to test the reactivity of the GLPL serum by western blots. As shown in Figure 3A, the human autoantibody reacts in HeLa cell extracts with a 135 kD polypeptide in a titration assay (lanes 1–3, Figure 3A). Similar result was observed in blots with CHO cell extracts (data not shown), although other polypeptides of 91, 38, and 16 kd were also observed when the blots were overexposed. Putatively, some of these polypeptides represent the autoantigen(s) responsible for the IF pattern observed in Figures 1 and 2. Based on these IF results, we decided to test for reactivity of the GLPL serum by immunoblot against a chromosomal passenger protein complex. We used a protein extract (Figure 3B lane 1) containing three of the major centromere passenger proteins (INCENP, Aurora B and Survivin) expressed in Sf9 culture insect cells by the baculovirus system [11]. The immunoblot clearly showed a strong reactivity (asterisk in Fig. 3B, lane 2) to the expected molecular weight for the expressed INCENP component of the chromosomal passenger complex (up arrow in Fig. 3B, lane1). No reactivity was observed in the same blot against the other two components of the complex present in the protein extract (two lower arrows in Fig. 3B, lane1). A blot control against the baculovirus expressed protein complex with a specific anti-INCENP serum shows the mobility of the INCENP protein component (asterisk in Fig. 3B lane 3). Figure 3, clearly show that human GL autoantibody and anti-INCENP serum recognized the same protein on blots. From this result it can be concluded that INCENP protein represents a major autoantigen in GLPL syndrome.

It is known that most of the anti-nuclear autoimmune sera (ANAs) in humans are directed against macromolecular complexes of proteins or protein-nucleic acid structures [12]. The centromere per se is a target for autoimmune diseases including scleroderma and CREST syndrome [13], where the major centromeric autoantigens described are CENP-A, CENP-B and CENP-C [14-16]. If the GLPL autoantibody used in this report also recognizes some other centromere antigens in addition to the INCENP protein, or some other component of the mitotic machinery, is a possibility that could not excluded, and more intensive experimentation will be required. Studies by immunoprecipitation and screenings of expression libraries are underway and could serve to test for that possibility.

INCENP is a major component of the centromere during several phases of the mitotic cell cycle where it plays an essential role in chromosome segregation and participates in the mechanisms regulating mitosis [9,10]. Depletion of INCENP by RNAi results in abnormal chromosome structure and defects in central spindle formation and cytokinesis [5]. The relationship between autoimmunity against INCENP and GLPL disease could be just fortuitous as for many other autoimmune responses in humans. However the levels of autoantibodies are good markers for monitoring the behavior of many human autoimmune diseases [12].

The GLPL syndrome represents a rare disease and documentation is scarce [2,3]. Our report represents the first evidence for autoimmunity in GLPL syndrome. By selecting a significant number of GLPL patients it may be possible to reach conclusions about the prevalence and clinical significance of the autoimmune response in this disease.

## References

[B1] Ghislain PD, Van Eeckhout P, Ghislain E (2003). Lassueur-Graham Little-Piccardi syndrome: 20-year follow-up. Dermatology.

[B2] Viglizzo G, Verrini A, Rongioletti F (2004). Familial Lassueur- Graham- Little- Piccardi syndrome. Dermatology.

[B3] Crickx B, Blanchet P, Grossin M, Belaich S (1990). Lassueur-Graham-Little syndrome. 2 cases. Ann Dermatol Venereol.

[B4] Bianchi L, Paro Vidolin P, Piemonte P, Carboni I, Chimenti S (2001). Graham Little- Piccardi-Lassueur syndrome: effective treatment with cyclosporin A. Clin Exp Dermatol.

[B5] Vagnarelli P, Earnshaw WC (2004). Chromosomal passengers: the four-dimensional regulation of mitotic events. Chromosoma.

[B6] Carvalho A, Carmena M, Sambade C, Earnshaw WC, Wheatley SP (2003). Survivin is required for stable checkpoint activation in taxol-treated HeLa cells. J Cell Sci.

[B7] Wheatley AP, Carvalho A, Vagnarelli P, Earnshaw WC (2001). INCENP is required for proper targeting of survivin to the centromeres and the anaphase spindle during mitosis. Curr Biol.

[B8] Honda R, Korner R, Nigg EA (2003). Exploring the functional interactions between aurora B, INCENP and survivin in mitosis. Mol Biol Cell.

[B9] Towbin H, Staehelin T, Gordon J (1979). Electrophoretic transfer of proteins from polyacrylamide gels to nitrocellulose sheets: procedure and some applications. Proc Natl Acad Sci USA.

[B10] Adams RR, Carmena M, Earnshaw WC (2001). Chromosomal passengers and the (aurora) ABCs of mitosis. Trends Cell Biol.

[B11] Gassmann R, Carvalho A, Henzing J, Ruchaud S, Hudson DF, Honda R, Nigg EA, Gerloff DL, Earnshaw WC (2004). Borealin; a novelchromosomal passenger required for stability of the bipolar mitotic spindle. J Cell Biol.

[B12] Tan EM (1989). Antinuclear antibodies: diagnostic markers for autoimmune diseases and probes for cell biology. Adv Immunol.

[B13] Moroi Y, Peebles C, Fritzler MJ, Steigerwald MJ, Tan EM (1980). Autoantibody to centromere (kinetochore) in scleroderma sera. Proc Natl Acad Sci USA.

[B14] Earnshaw WC, Sullivan KF, Machain PS, Cooke CA, Kaiser DA, Pollard TD, Rothfield NF, Cleveland DW (1987). Molecular cloning of cDNA for CENP-B, the major human centromere autoantigen. J Cell Biol.

[B15] Iwai T, Muro Y, Sugimoto K, Matsumoto Y, Ohashi M (1996). Clinical features of antibodies associated with anti-centromere antibodies. Clin Exp Immunol.

[B16] Muro Y, Yamada T, Himeno M, Sugimoto K (1998). cDNA cloning of a novel autoantigen targeted by a minor subset of anti-centromere antibodies. Clin Exp Immunol.

